# Access to episodic primary care: a cross-sectional comparison of walk-in clinics and urgent primary care centers in British Columbia

**DOI:** 10.1017/S1463423623000580

**Published:** 2023-11-28

**Authors:** Mary A. McCracken, Ian R. Cooper, Michee-Ana Hamilton, Jan Klimas, Cameron Lindsay, Sarah Fletcher, Morgan Price, Lindsay Hedden, Rita K. McCracken

**Affiliations:** 1 Innovation Support Unit, Faculty of Medicine, University of British Columbia, Vancouver, BC, Canada; 2 Department of Family Practice, University of British Columbia, Vancouver, BC, Canada; 3 Faculty of Health Sciences, Simon Fraser University, Vancouver, BC, V6T 2A1, Canada

**Keywords:** after-hours care, delivery of health care, health services accessibility, primary health care

## Abstract

**Aim:**

This study aimed to identify publicly reported access characteristics for episodic primary care in BC and provided a clinic-level comparison between walk-in clinics and UPCCs.

**Background:**

Walk-in clinics are non-hospital-based primary care facilities that are designed to operate without appointments and provide increased healthcare access with extended hours. Urgent and Primary Care Centres (UPCCs) were introduced to British Columbia (BC) in 2018 as an additional primary care resource that provided urgent, but not emergent care during extended hours.

**Methods:**

This cross-sectional study used publicly available data from all walk-in clinics and UPCCs in BC. A structured data collection form was used to record access characteristics from clinic websites, including business hours, weekend availability, attachment to a longitudinal family practice, and provision of virtual services.

**Findings:**

In total, 268 clinics were included in the analysis (243 walk-in clinics, 25 UPCCs). Of those, 225 walk-in clinics (92.6%) and two UPCCs (8.0%) were attached to a longitudinal family practice. Only 153 (63%) walk-in clinics offered weekend services, compared to 24 (96%) of UPCCs. Walk-in clinics offered the majority (8,968.6/ 78.4%) of their service hours between 08:00 and 17:00, Monday to Friday. UPCCs offered the majority (889.3/ 53.7%) of their service hours after 17:00.

**Conclusion:**

Most walk-in clinics were associated with a longitudinal family practice and provided the majority of clinic services during typical business hours. More research that includes patient characteristics and care outcomes, analyzed at the clinic level, may be useful to support the optimization of episodic primary healthcare delivery.

## Background

Canada’s essentially universal healthcare coverage ensures that a usual source of primary care is available to most of the population; however, issues with a shortage of care providers persist in some provinces more than in others (Xu, 2022; Kiran & Pham, 2023). In contrast to longitudinal primary care, episodic primary care facilities generally operate without appointments, provide urgent, but not emergent, care, and increase healthcare access with extended hours.

Walk-in clinics in Canada offer community-based, episodic care for patients with minor illnesses and injuries (Miller et al., 1989; Jones, 2000; Matthewman et al., 2021). As non-hospital-based facilities, they operate without appointments, with some providing after-hours care. Operating in British Columbia (BC) since 1986 (Medical Staff Writer, 1987), they were initially marketed as a convenient alternative to the emergency department for after-hours care (Blaine, 1988). In BC, they are typically funded via fee-for-service physician remuneration (Mitra et al., 2021) and have the same College of Physicians and Surgeons practice standard as longitudinal primary care clinics (College of Physicians & Surgeons of British Columbia, 2021). However, some studies have found that continuity of care in practice standards for walk-in clinics is variable and may not establish the beneficial longitudinal relationships between patients and providers that have been shown to reduce mortality (Pereira Gray et al., 2018; Lofters et al., 2023). Across Canada, provincial and territorial Colleges’ practice standards vary. The function and role of walk-in clinics may also vary provincially, shifting based on unmet regional needs. In 2018, Urgent and Primary Care Centres (UPCCs) were introduced to BC to provide episodic urgent, but not emergent, primary care services during extended hours (BC Ministry of Health & Deputy Ministry of Health, 2019). UPCCs receive funding via their regional Health Authority (HA) and offer allied healthcare services, and physicians are paid via an alternative payment plan (Mitra et al., 2021). A family doctor shortage has been reported in BC (Lee, 2000) since 2000 and throughout Canada (Hedden et al., 2017; Xu, 2022). Approximately 15–30% of BC residents are not attached to longitudinal primary care (Statistics Canada, 2020; Kiran & Pham, 2023).

Recent research from both North American and non-North-American primary care settings, including systematic reviews, retrospective cohort studies, and cross-sectional survey research, has determined that access to longitudinal primary care services after-hours has variable impact on the volume of emergency department visits (van den Berg et al., 2016; Kiran et al., 2018; Hong et al., 2020; Baughman et al., 2021). The impact of episodic primary care on emergency department usage in Canada and around the globe is understood even less. Some studies have found that urgent care facilities that are not in hospitals for more acute issues may increase costs, as found in a cost analysis conducted in America by Wang and colleagues (Wang et al., 2021); a systematic review of international literature conducted in 2018 found that urgent care facilities had variable effects on emergency department crowding (Morley et al., 2018). While episodic care is used by both attached and unattached patients, walk-in clinics have been criticized for disrupting continuity of care that has been shown to decrease mortality, hospitalizations, and healthcare costs (Jones, 2000; Bazemore et al., 2018; Pereira Gray et al., 2018; Baker et al., 2020). Other outcomes for walk-in clinics examined in the literature include patient demographics, the most common conditions associated with patient visits, and patient value for continuity of care. Salisbury and Munro’s review of the international literature for walk-in clinics found that females, young adults, and people who were employed were over-represented in walk-in clinic patient demographics (Salisbury & Munro, 2003). Patients most often visited walk-in clinics for skin disorders, musculoskeletal conditions, and respiratory tract infections, and many patients were indifferent about the lack of continuity of care.

Within the last decade, few published studies described clinic numbers, characteristics, or effects of episodic primary care in Canada (Salisbury & Munro, 2003; Lapointe-Shaw et al., 2023). An accurate baseline description is important to monitor changes over time and to understand how episodic primary care impacts longitudinal care. We sought to describe and compare publicly reported access characteristics between two modalities of episodic primary care, walk-in clinics and urgent and primary care facilities in BC, including clinic service hours, after-hours availability, association with longitudinal primary care, booking methods, and appointment types. We will also describe regional variations in number of clinic hours available per week, per 100 000 population in BC.

## Methods

### Study design and setting

This is a cross-sectional study of access characteristics of outpatient clinics providing episodic primary care in BC, Canada. Access to care is understood to comprise both the availability and utilization of services, and the current analysis focuses on the availability of services. BC has a single-payer healthcare insurance program, with no direct-to-patient charges for primary care services delivered at walk-in clinics or UPCCs (Hutchison et al., 2011). Physician payment is largely by fee-for-service, with only a few clinics using a capitated model (Mitra et al., 2021), that includes negation to the physician’s payment if their patients go to walk-in clinics (Glazier et al., 2019).

### Data collection and sources

We used publicly available lists of clinic information including the BC Ministry of Health’s Walk-in Clinic List (Walk-in Clinic List) version: April 2021 (Ministry of Health, 2021), and the Urgent and Primary Care Clinic (UPCC) List, version: November 2021 (BC Ministry of Health, 2021). The Walk-in Clinic List is maintained by the Ministry of Health and reviewed for completeness by a data steward at the BC Data Catalogue (Ministry of Health - HealthLinkBC, 2020). The listing includes clinic name, website, phone number, street address, and additional data. The UPCC list (BC Ministry of Health, 2021) is regularly updated on HealthLink BC, and the public-facing source of non-emergency health information and advice is maintained by the BC Ministry of Health. This site provides the names of UPCCs, organized by HA, and a link to a clinic-specific website (BC Ministry of Health, 2021).

Both lists were reviewed for duplication. If either list did not include a website address, the clinic and street address were searched via Google. For clinics with no self-managed website found in a Google search, information would be extracted from one of the public, aggregate walk-in clinic websites, searched in order: Medimap (*Medimap*, n.d.), SkiptheWaitingRoom (*Skip The Waiting Room*, n.d.), Pathways (Pathways, n.d.), and Cortico (Cortico Health Technologies, n.d.).

A structured data collection form was used to review clinics’ websites for the variables of interest. Author [MAM] collected data from walk-in clinic websites between May and June 2021 and from UPCC websites twice in June and November 2021, to account for 13 new UPCCs that opened during the study period.

Population data by Health Authority (HA) was retrieved from the BC government population estimates and projections website (BC Stats, Government BC, 2021). Oversight of access to and quality of healthcare services in BC is delivered by 5 different geographically defined HAs and the First Nations Health Authority (FNHA) which is responsible for managing health programs and services for First Nations people in the entire province (Ministry of Health, n.d.). Clinic service hours per 100 000 people are reported because health system monitoring and funding is directed to HAs (Ministry of Health, 2020).

### Variables of interest

#### Clinic service hours

Regular business hours were defined as 08:00-17:00, Monday to Friday. After-hours access was defined as either patient services provided Monday to Friday, before 08:00, after 17:00, or after 20:00, or any patient services available on weekends (Saturday and/or Sunday).

#### Association with other primary care services

If a walk-in clinic website stated they provided attachment for longitudinal care or specifically described an association with a separate longitudinal clinic, it was associated with a longitudinal family practice. This information was collected to help understand the possible relationship between longitudinal and episodic primary care provision. The presence of a co-located pharmacy was recorded as a proxy measure for the presence of primary care services in addition to family doctor consultation and may be present for both walk-in clinics and UPCCs. This was determined if an adjacent pharmacy was listed on their website or observed by author [Author initials 1] through visualization on Google Maps (*Google Maps*, n.d.).

#### Appointment booking options and availability of virtual care options

Appointment booking information was collected from clinics’ websites. Ability to ‘walk-in’ was determined if the website said patients did not need to be previously known or did not require a booked appointment to receive care. Types of ‘walk-in appointments’ included walk-in only, phone booking, online and email bookings. Listed options for telephone or video appointments were identified as virtual services. A change in access to services due to COVID was determined by the clinic’s website mentioning that walk-in services were no longer being provided or requesting that patients call in before coming to the clinic.

### Data analysis

We used a chi-square test to compare categorical variables, stratified by HA and clinic type (walk-in clinics *vs.* UPCCs), and calculated descriptive statistics using the IBM Statistical Package for the Social Sciences software (V27.0; IBM). We accessed publicly available information, and per article 2.2 of the Tri-Council Policy Statement 2 (2018) did not require review by an ethics board (Government of Canada, I. A. P. on R. E, 2019).

## Results

Our analysis included 268 clinics, including 243 walk-in clinics and 25 UPCCs. The Walk-in Clinic List (April 2021) listed 256 clinics. Thirteen clinics were excluded, 12 because they were permanently closed and one did not list any service hours. Eighty walk-in clinics did not list a website, 42 walk-in clinic websites were found via a Google search, and for the remaining 38 walk-in clinics, data were collected from one of the aforementioned aggregate websites (*Medimap*, n.d.),(*Skip The Waiting Room*, n.d.),(Pathways, n.d.),(Cortico Health Technologies, n.d.).

### Clinic service hours

In a typical week in BC, 13 094 h of episodic primary care are available (Table 1). Fourteen (5.8%) walk-in clinics provide patient services before 08:00, compared to no UPCCs. Sixteen (6.6%) walk-in clinics offer services after 17:00, compared to 22 (88%) UPCCs, and 153 (63%) walk-in clinics are open on weekends, compared to 24 (96%) UPCCs. The majority (8,968.6/ 78.4%) of walk-in clinics’ service hours were between 08:00 and 17:00, Monday to Friday. UPCCs offered the majority (889.3/ 53.7%) of their service hours after 17:00. Only 145 (59.7%) walk-in clinics, and none of the UPCCs listed the names or number of doctors who worked there (median 5 [interquartile range (IQR) = 3.7]).

**Table 1.** Clinic service hours for walk-in clinics and Urgent Primary Care Centres in British Columbia^1^

Health Authority^a^	Total Hours Open Per Week^b^, hours	Hours Open Per Week median (IQR)	Hours Open Before 08:00^c^ *n* (%)	Hours Open After 17:00^c^ *n* (%)	Hours Open After 20:00^c^ *n* (%)	Hours Open on Weekend^d^ *n* (%)	Number of MDs Listed as Working at Clinic^e^ median (IQR)
Interior							
Walk-in clinic (*n* = 26)	1208.2	44.5 (IQR 39,53)	5 (0.4%)	160 (13.2%)	5 (0.4%)	150 (12.4%)	4.5 (IQR 3,8)
UPCC (*n* = 6)	387.0	77 (IQR 35,84)	0 (0.0%)	107.5 (27.8%)	17.5 (4.5%)	122 (31.5%)	n/a
Fraser							
Walk-in clinic (*n* = 97)	4353.9	44 (IQR 38,53)	37.5 (0.9%)	297 (6.8%)	15 (0.3%)	482 (11.1%)	4.0 (IQR 3,7)
UPCC (*n* = 6)	335.0	52.5 (IQR 41,77)	0 (0.0%)	90 (26.9%)	15 (4.5%)	110 (32.8%)	n/a
Vancouver Coastal							
Walk-in clinic (*n* = 71)	3595.3	50.5 (IQR 40,59)	12.5 (0.3%)	309 (8.6%)	17 (0.5%)	496 (13.8%)	5.0 (IQR 3,8)
UPCC (*n* = 5)	408.0	92 (IQR 66,92)	0 (0.0%)	120 (29.4%)	45 (11.0%)	108 (26.5%)	n/a
Vancouver Island							
Walk-in clinic (*n* = 36)	1782.8	45 (IQR 35,62)	10.5 (0.6%)	167 (9.4%)	26.5 (1.5%)	272.5 (15.3%)	6 (IQR 4,10)
UPCC (*n* = 6)	419.0	83.5 (IQR 46,84)	0 (0.0%)	65 (15.5%)	5 (1.2%)	104 (24.8%)	n/a
Northern							
Walk-in clinic (*n* = 13)	536.5	40 (IQR 36,45)	0 (0.0%)	35 (6.5%)	5 (0.9%)	35 (6.5%)	2 (IQR 0,5)
UPCC (*n* = 2)	107.0	53.5 (IQR n/a)	0 (0.0%)	35 (32.7%)	5 (4.7%)	27 (25.2%)	n/a
BC Total							
Walk-in clinic (*n* = 243)	11437.6	45 (IQR 39,56)	65.5 (0.6%)	968 (8.5%)	68.5 (0.6%)	1439.3 (12.5%)	5 (IQR 3,7)
UPCC (*n* = 25)	1656.0	77 (IQR 48,84)	0 (0.0%)	417.5 (25.2%)	87.5 (5.3%)	471 (28.4%)	n/a

a British Columbia is divided into 5 geographic health authorities.

b Hours available for patient services as collected from clinic websites.

c Monday to Friday hours only.

d Saturday to Sunday hours only.

e Count of family physician names listed as providing patient services on clinic website.

### After-hours availability, association with longitudinal primary care, booking methods, and appointment types

Table 2 compares primary care access characteristics between walk-in clinics and UPCCs and shows significant differences between the clinic types, including being associated with longitudinal practice, appointment booking, and virtual appointment options. Most walk-in clinics in BC (92.6%) are operated as a part of, or adjacent to, a longitudinal family practice and offer the majority of service hours during weekday business hours (08:00-17:00). Further, they offer phone (99.6%), online booking (46.9%), and virtual appointments (73.3%) at significantly higher rates than UPCCs (44%, 0% and 20% respectively).

**Table 2.** Access characteristics of walk-in clinics and Urgent and Primary Care Centres (UPCCs)

Access Characteristic	Walk-in clinics (*n* = 244) *n* (%)	UPCCs (*n* = 25) *n* (%)	X sq	*P*-value
Clinic is associated with a longitudinal primary care practice	225 (92.6%)	2 (8.0%)	125.18	<.001
Patient may walk-in^a^	223 (91.8%)	20 (80.0%)	3.712	0.054
Notice of change to walk-in access service due to COVID-19^b^	108 (44.4%)	3 (12.0%)	9.834	0.002
Appointment booking^c^				
Walk-in only	1 (0.4%)	14 (56.0%)	132.568	<.001
Phone	242 (99.6%)	11 (44.0%)	143.580	<.001
Online	114 (46.9%)	0 (0.0%)	20.410	<.001
Email Bookings	19 (7.8%)	0 (0.0%)	2.104	0.147
Virtual appointments available^d^	178 (73.3%)	5 (20.0%)	29.680	<.001
Phone appointments	176 (72.4%)	5 (20.0%)	28.419	<.001
Video appointments	136 (56.2%)	3 (12.0%)	17.736	<.001
Pharmacy attached to clinic	62 (25.5%)	3 (12.0%)	2.254	0.133
Only open between 08:00-17:00, M-F	65 (26.7%)	1 (4.0%)	6.32	0.012

a Patient does not need to be previously known nor have a booked appointment with clinic.

b Website included a description of how patient services access has changed due to COVID.

c Methods by which a patient can book an appointment with the clinic.

d Virtual appointments are available at this location.

**Table 3.** Practice guidelines for walk-in clinics and episodic care across Canada compared to British Columbia

Province	Practice Guidelines										
	Has a document or policy for walk-in clinics or episodic care	States that physicians are expected to meet the professional standard of practice	Must collect and document a thorough health history and current health problems	Must identify PCP^*^ and provide summary of episodic interaction	Must inform patient that the regulated member will not provide ongoing care	Patients who attend clinic repeatedly (and do have a PCP) must be assumed to receive primary health care from that clinic	Clinic has a designated Medical Director	Physicians must ensure appropriate follow-up and continuity of care	Must ensure after-hours^**^ coverage is available	Must have on-site access to provincial prescribing records and/or EHR^***^ for documentation	Must review medical record and history prior to prescribing controlled substances
British Columbia^^3^,^11^^	X	X	X	X	X	X	X	X	X	X	X
Alberta^^1^,^12–^ ^14^^	X		X	X	X	X		X	X	X	
Saskatchewan^^6^,^15^^	X	X	X	X		X		X	X		X
Manitoba^^2^^			X	X				X			
Ontario^^4^,^16^^	X	X	X	X	X			X	X		
Quebec^****^^^17^,^18^^		X	X					X			
New Brunswick^^8^^	X	X	X	X	X			X		X	
Newfoundland and Labrador^^9^^		X	X	X		X	X	X			
Prince Edward Island^^5^^	X	X	X	X	X			X	X	X	X
Nova Scotia^^7^^	X		X	X	X			X			X
Yukon^^10^^	X		X	X	X			X			X
Northwest Territories^*****^											
Nunavut^******^											

* PCP: primary care provider.

** After-hours coverage was interpreted as the healthcare professionals ensuring the patients could go to a different healthcare facility when their walk-in clinic was closed.

*** EHR: electronic health record.

**** College de medicines du Quebec does not have specific guidelines for walk-in clinics as per personal correspondence with the Investigations Director of the College.

***** No relevant information could be found from the Northwest Territories Standards of Practice or their Health and Social Services government website.^^19^^ They did not respond to our general inquiry surrounding walk-in clinics and episodic care.

****** As per personal correspondence with the Charge Physician of Nunavut, it has been confirmed that there are no walk-in clinics in the territory and this role is fulfilled by the health centers and the emergency department in Iqaluit.

### Geographic variation in availability of clinic service hours

**Figure 1.**
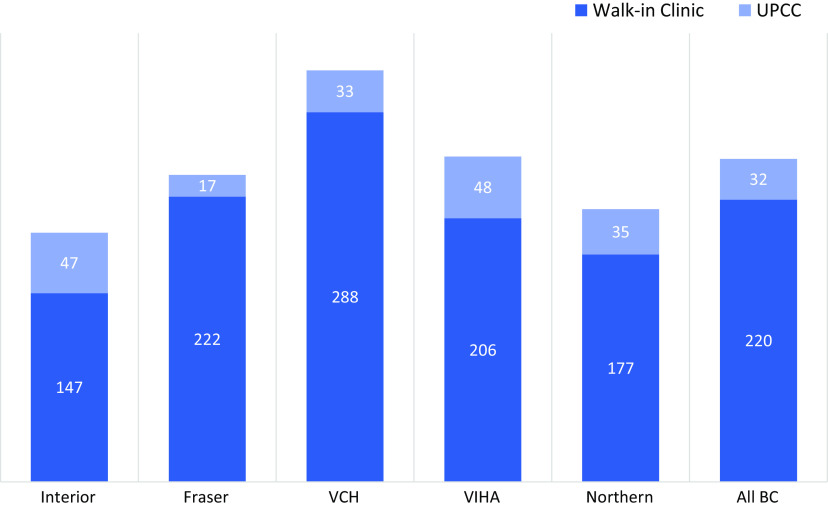
Variation in Number of Clinic Service Hours Available Per Week, Per 100 000 Population, by Health Authority. British Columbia is divided into 5 geographic health authorities: Interior, Fraser, Vancouver Coastal (VCH), Vancouver Island (VIHA), and Northern Population estimates for each health authority were collected from BCStats BC Populations Estimates and Projections (BC Stats, Government BC, 2021).

Figure 1 describes and compares the total clinic service hours per 100 000 population between walk-in clinics and UPCCs across all five geographic HAs. Interior and Northern Health Authorities were found to have lower hours per 100 000, than Fraser, Vancouver Coastal, and Vancouver Island Health Authorities. In all regions, walk-in clinics provide the majority of available hours for episodic primary care.

## Discussion

The study described and compared access characteristics of two types of episodic primary care in BC using publicly available data. The majority of episodic care is provided by walk-in clinics, and clinic characteristics varied for walk-in clinics versus UPCCs.

Almost all walk-in clinics in BC (92.6%) are operated at the same site as a longitudinal family practice, and the majority of services are provided during regular business hours (78.4%). If the shared-site walk-in clinics were serving as adjuncts to longitudinal care for patients ‘attached’ to the longitudinal practice, we would expect that after-hours services would be more available for attached patients whose health issues occur outside of regular hours (Salisbury & Munro, 2003; Chapman et al., 2004). Instead, the after-hour services are potentially being used by patients unattached to a longitudinal practice. The episodic services are potentially being used by people unattached to a longitudinal practice (Prince, 2016; Glazier et al., 2019). Therefore, it is possible that these same-site episodic services are more financially rewarding to offer than an expansion of longitudinal services. The mechanisms that enable increased access to episodic care, potentially at the expense of longitudinal care access, are worrisome for a number of reasons: (i) there is evidence that people experiencing barriers to health equity are over-represented among the total population of those unattached to longitudinal care (Marshall et al., 2017; Lavergne et al., 2022); (ii) episodic primary care has been observed to have fewer patient and system-level benefits (Baker et al., 2020; Lofters et al., 2023); and (iii) ultimately to be more costly than longitudinal primary care (Bazemore et al., 2018). In this region, where access is inadequate and there are shortages of primary care providers, researchers should examine how episodic care acts as a barrier to improving access to longitudinal care.

We found significant differences in appointment booking, service hours per 100 000 population, and virtual appointment options between UPCCs and walk-in clinics. Firstly, some large areas of less densely populated portions of the province, which were covered by the Interior and Northern Health Authorities, had lower hours per 100 000 population than Fraser, Vancouver Coastal, and Vancouver Island Health Authorities, which each contain at least one large metropolitan area. Secondly, walk-in clinics surpassed UPCCs in phone or online booking and virtual visits, which we speculate may be occurring due to their higher nimbleness in adapting to digital care. However, the impact of episodic primary care (including digitalized episodic primary care) on longitudinal care availability has not been fully characterized; moreover, a recent retrospective study, literature review, and cost analysis, which looked at costs and emergency department utilization in other countries, have yielded mixed results (Patwardhan et al., 2012; Ismail et al., 2013; Wang et al., 2021). In line with the findings of our study, previous international studies, and systematic reviews, we urge careful consideration of local context before creating policies and increasing episodic after-hours access more widely (Hong et al., 2020; Baughman et al., 2021; McKay et al., 2022).

Episodic primary care accessibility varies geographically in BC. BC operates using the Health Authority system, where each HA is responsible for identifying and meeting their populations’ health needs (Ministry of Health, n.d.). Primary care attachment, continuity of care, and after-hours service provision may differ according to need across Health Authorities, or some areas may have insufficient resources available. The authors are unaware of any published studies that estimate the ‘appropriate’ amount of episodic primary care for a region.

### Limitations

The findings of our study are specific to a single region in Canada (Table 3) which limits their generalizability to other jurisdictions. Limitations of this study include the reliance on publicly available data from a single region in Canada that may not be updated, may not reflect actual practices, or may not be applicable to other jurisdictions. Secondly, this study could only measure access via clinics’ business hours, methods of booking appointments, adjacency to a pharmacy, and availability of virtual services. The number of clinicians at work during opening hours was reported inconsistently and none reported the volume of patients seen per shift. Consequently, our data cannot be used to indicate the daily patient volume which would be a more ideal report of service capacity.

### Conclusion

Using publicly available data, we have described episodic primary care access across BC which may be useful to establish a baseline measure to inform future research, evaluation, or policy development of episodic primary care capacity and impact. This approach has the potential to be used more widely to understand the phenomenon of episodic primary care in other geographic areas. The finding of regional variations in the number of episodic primary care clinic service *hours available per 100 000 population* is a potential proxy measure for under-/overservicing with episodic care or may be reflective of unique local context. Most walk-in clinics are associated with a longitudinal family practice and provide services during typical business hours. Future studies that include patient care information and other outcomes from administrative data and a more comprehensive study of access characteristics analyzed at the clinic level would be useful to support the optimization of episodic and longitudinal primary healthcare delivery.

## Notes

1. College of Physicians and Surgeons Alberta (2015) *Standards of Practice: Episodic Care* [Internet]. 2015 June. Retrieved 5 May 2021 from https://cpsa.ca/search/practicestandards/

2. College of Physicians and Surgeons Manitoba (2018) *College of Physicians and Surgeons of Manitoba Standards of Practice Regulation* [Internet]. 2018 December. Retrieved 7 May 2021 from https://cpsm.mb.ca/laws-and-policies/standards-of-practice-of-medicine

3. College of Physicians & Surgeons of British Columbia (2018) *Primary Care Provision in Walk-in, Urgent Care, and Multi-Physician Clinics* [Internet]. CPSBC; 2019 June. Retrieved 27 April 2021 from https://www.cpsbc.ca/files/pdf/PSG-Walk-In-Urgent-Care-Multi-Physician-Clinics.pdf

4. College of Physicians and Surgeons Ontario (2019) *Walk-in Clinics* [Internet]. 2019 September. Retrieved 7 May 2021 from https://www.cpso.on.ca/Physicians/Policies-Guidance/Policies/Walk-in-Clinics

5. College of Physicians and Surgeons of Prince Edward Island (2019) *Guideline on Standard of Care Walk-In Clinics* [Internet]. 2019 November. Retrieved 7 May 2021 from https://cpspei.ca/publications/policies/

6. College of Physicians and Surgeons of Saskatchewan (2020) *Clinics that Provide Care to Patients who are not Regular Patients of the Clinic* [Internet]. 2020 January. Retrieved 5 May 2021 from https://www.cps.sk.ca/imis/CPSS/Legislation__ByLaws__Policies_and_Guidelines/Legislation_Content/Policies_and_Guidelines_Content/Clinics_that_Provide_Care_to_Patients_who_are_not_Regular_Patients_of_the_Clinic.aspx

7. College of Physicians and Surgeons of Nova Scotia (2020) *Professional Standards Regarding the Responsibilities of Physicians Working in Walk-in Clinic*s [Internet]. 2020 March. Retrieved 7 May 2021 from https://cpsns.ns.ca/resource/physicians-working-in-walk-in-clinics/

8. College of Physicians and Surgeons of New Brunswick (2021) *Guidelines- Walk-In Clinics* [Internet]. Retrieved 7 May 2021 from https://cpsnb.org/en/physicians/professional-standards2/professional-standards/450-walk-in-clinics

9. College of Physicians and Surgeons of Newfoundland and Labrador (2021) *Standard of Practice- Continuity of Care* [Internet]. 2021 March. Retrieved 7 May 2021 from https://www.cpsnl.ca/WEB/CPSNL/Standards_of_Practise/Policies__Guidelines__Advisories/CPSNL/Policies/Policies__Guidelines_and_Advisories.aspx?hkey=392e3ee4-8d3e-49d4-920b-369d54862c60

10. Yukon Medical Council (2021) *Episodic Care* [Internet]. Retrieved 7 May 2021 from http://www.yukonmedicalcouncil.ca/standards.html

11. College of Physicians & Surgeons of British Columbia (2021) *Medical Records, Data Stewardship and Confidentiality of Personal Health Information* [Internet]. Retrieved  1 April 2021 from https://www.cpsbc.ca/files/pdf/PSG-Medical-Records.pdf

12. College of Physicians & Surgeons of Alberta (2016) *Standards of Practice: Patient Record Retention* [Internet]. Retrieved  1 April 2021 from https://cpsa.ca/wp-content/uploads/2020/05/Patient-Record-Retention.pdf

14. College of Physicians & Surgeons of Alberta (2019) *Advice to the Profession: Episodic Care* [Internet]. Retrieved  1 April 2021 from https://cpsa.ca/wp-content/uploads/2020/06/AP_Episodic-Care.pdf

15. College of Physicians and Surgeons of Saskatchewan (2021) *Medical Practice Coverage* [Internet]. Retrieved 16 August 2021 from https://www.cps.sk.ca/imis/CPSS/Legislation__ByLaws__Policies_and_Guidelines/Legislation_Content/Policies_and_Guidelines_Content/Medical_Practice_Coverage.aspx

16. College of Physicians and Surgeons Ontario (2019) *Availability and Coverage* [Internet]. Retrieved 16 August 2021 from https://www.cpso.on.ca/Physicians/Policies-Guidance/Policies/Availability-and-Coverage

17. College des Medicines (2017) Code of ethics of physicians. 20. Retrieved 13 July 2023 from https://canlii.ca/t/7rgz

18. College des Medicines (2013) Record keeping by physicians in non-hospital settings. 2013 Jun;47. Retrieved 13 July 2023 from https://canlii.ca/t/zx1

19. Northwest Territories Health and Social Services Professionals (2015) *Community Health Core Service Standards and Protocols* [Internet]. Government of the Northwest Territories; 2015 February. Retrieved 7 May 2021 from https://www.hss.gov.nt.ca/professionals/en/services/policies-and-guidelines-standards-and-manuals
